# PanKA: Leveraging population pangenome to predict antibiotic resistance

**DOI:** 10.1016/j.isci.2024.110623

**Published:** 2024-08-02

**Authors:** Van Hoan Do, Van Sang Nguyen, Son Hoang Nguyen, Duc Quang Le, Tam Thi Nguyen, Canh Hao Nguyen, Tho Huu Ho, Nam S. Vo, Trang Nguyen, Hoang Anh Nguyen, Minh Duc Cao

**Affiliations:** 1 Center for Applied Mathematics and Informatics, Le Quy Don Technical University, Hanoi, Vietnam; 2 Center for Biomedical Informatics, Vingroup Big Data Institute, Hanoi, Vietnam; 3 AMROMICS JSC, Vinh, Nghe An, Vietnam; 4 Faculty of IT, Hanoi University of Civil Engineering, Hanoi, Vietnam; 5 Oxford University Clinical Research Unit, Hanoi, Vietnam; 6 Bioinformatics Center, Institute for Chemical Research, Kyoto University, Kyoto, Japan; 7 Department of Medical Microbiology, The 103 Military Hospital, Vietnam Military Medical University, Hanoi, Vietnam; 8 Department of Genomics & Cytogenetics, Institute of Biomedicine & Pharmacy, Vietnam Military Medical University, Hanoi, Vietnam

**Keywords:** genomics, bacteriology, machine learning

## Abstract

Machine learning has the potential to be a powerful tool in the fight against antimicrobial resistance (AMR), a critical global health issue. Machine learning can identify resistance mechanisms from DNA sequence data without prior knowledge. The first step in building a machine learning model is a feature extraction from sequencing data. Traditional methods like single nucleotide polymorphism (SNP) calling and k-mer counting yield numerous, often redundant features, complicating prediction and analysis. In this paper, we propose PanKA, a method using the pangenome to extract a concise set of relevant features for predicting AMR. PanKA not only enables fast model training and prediction but also improves accuracy. Applied to the *Escherichia coli* and *Klebsiella pneumoniae* bacterial species, our model is more accurate than conventional and state-of-the-art methods in predicting AMR.

## Highlights

• We develop PanKA to leverage pangenome for predicting antimicrobial resistance
• PanKA enhances prediction accuracy and accelerates model training and prediction
• PanKA surpasses traditional and SOTA methods in predicting antimicrobial resistance

## Graphical abstract

**Figure:**
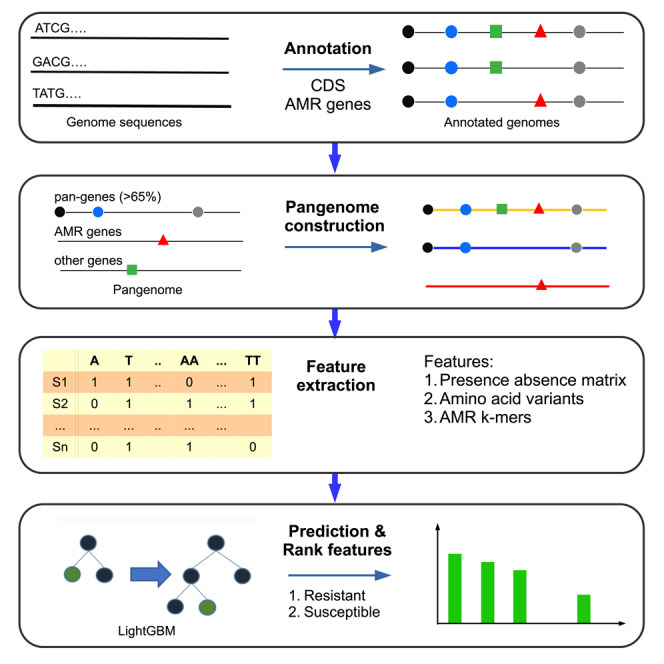

## Introduction

The discovery and development of antibiotics have played a vital role in the long history of fighting infectious diseases, but their widespread applications, if not inappropriate use, have also led to the emergence of drug-resistant bacterial strains.^1^ Virtually every antibiotic drug that has ever been developed is now associated with antimicrobial resistance (AMR).^2^ AMR is estimated to cause over 700,000 deaths per year, and this number could increase to 10 million by 2050 if without intervention.^3^ The World Bank has warned that uncontrolled AMR could result in annual financial costs of up to $3.4 trillion by 2030.^4^ Therefore, being able to accurately predict the AMR profiles and to prescribe the most effective antibiotic drugs to patients in a timely manner is of great significance.

Advances in DNA sequencing technologies in the last two decades have opened the opportunity to study the genomics mechanisms of AMR, giving rise to the development of computational methods to predict AMR from the sequencing of pathogenic bacterial genomes. Early methods for predicting AMR relied on direct association analysis, which involved detecting known resistance markers to determine resistance. However, this approach is only effective when the genetic markers of antibiotic resistance are well known, and typically they are not always available, especially for new antibiotic resistant strains. Moreover, this approach cannot take into account the possibility that these markers may have varying degrees of predictive power. Consequently, the development of AMR prediction models relying on supervised machine learning is increasingly common. The goal is to develop a prediction model that can infer the resistance profile of a new strain based on genomic features. This is achieved by training a machine learning model on a collection of genomes with known reference phenotypes. In addition, machine learning can detect new markers or combinations of markers, which is useful in situations where there is little or no prior knowledge of AMR mechanisms.^5^^,^^6^

The first step in developing machine learning models is feature engineering where a set of features are selected based on some domain knowledge. Those features are then used to train a machine learning model, such as support vector machines,^7^ neural networks,^8^ or gradient-boosted decision trees.^9^ Methods for feature extraction of bacterial sequence data can be divided into two paradigms: reference-free and reference-based approaches. In the reference-free approaches, the most prominent method is to count *k-mers* or to consider the presence and absence of *k-mers* in genomics or protein sequences. The main advantage of *k-mer* profiling is that it does not require aligning the genomes to a common reference sequence. This is particularly beneficial for species with rapidly changing genomes, where defining a common reference can be challenging. However, interpreting *k-mers* as genomic determinants is not always straightforward. The *k-mer* profile of the whole genomes inherently creates a very high dimensional feature space with strongly correlated variables leading to over-fitting the machine learning models.^10^^,^^11^^,^^12^

In the reference-based approach, single nucleotide polymorphisms (SNPs) are extracted by aligning bacterial genomes to the reference genome and are used as features to train the machine learning model.^7^^,^^13^ The SNP calling-based AMR prediction paradigm has a drawback due to its reliance on a reference genome, which is not easily defined for most bacteria species due to the plasticity of their genomes.^14^ Recently, the pangenome has been used as an alternative to the reference genome.^14^^,^^15^^,^^16^^,^^17^^,^^18^^,^^19^^,^^20^ The pangenome efficiently represents the complete genetic information of a species by collapsing homologous genes into clusters representing gene families, or genes for short. The genes present in the genomes of most strains are known as core genes, while those present in only some strains are known as accessory genes. The presence or absence of accessory genes in each genome was used as features to predict AMR.^9^^,^^21^^,^^22^ Several methods combine both sets of features, as well as other characteristics such as indels and years of isolation.^9^

To address the challenges posed by both reference-free and reference-based approaches, we develop PanKA (Pangenome and K-mer profile of AMR genes based model), a machine learning model that combines multiple types of AMR-relevant features extracted from the pangenome. PanKA trains a LightGBM^23^ model, a machine learning framework that utilizes decision trees to construct models. PanKA can extract various types of features, including the presence and absence of genes, the protein *k-mer* profile of AMR genes, and variant sites of core genes, in an efficient manner. By utilizing the most relevant features for AMR prediction, PanKA achieves higher prediction accuracy compared to current AMR prediction methods, which often rely on attributes that contain numerous irrelevant and redundant signatures. In addition, PanKA provides faster running times and offers better interpretability of the genomic determinants. We validate the proposed method, along with other existing feature extraction methods by training models to predict the AMR of two bacterial species, namely *Escherichia coli* and *Klebsiella pneumoniae* on 20 antibiotics. The results indicate that our proposed method is more accurate than conventional approaches using k-mers profiles as well as the current state-of-the-art classification method that exploits the pangenome.

## Results

### Overview of algorithm

**Figure 1:**
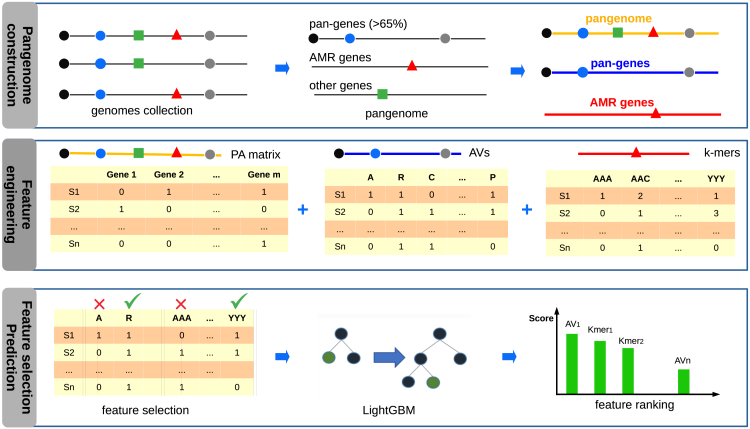
Overview of the PanKA algorithm *(top*—*Pangenome construction)* A pangenome is constructed from a collection of genomic sequences of strains. Pan-genes contain gene clusters comprising at least 65% of all strains, and AMR genes refer to the gene clusters with at least one AMR gene. *(middle*—*Feature engineering)* PanKA extracts the presence and absence matrix (PA matrix) from the pangenome, the amino acid variants (AVs) from the pan-genes, and k-mer profiles from the AMR gene clusters. *(bottom*—*Feature selection & prediction)* PanKA performs feature selection and prediction using the LightGBM model. The LightGBM model ranks the features according to their relevance in the prediction and extracts meaningful features from the data.

PanKA trains a machine learning model for a bacterial species using the genome sequences of a set of its strains as the training data. First, these genome sequences are annotated using Prokka (v1.14.5)^24^ to identify protein-coding sequences (CDSs) and AMR genes. Subsequently, PanKA constructs the species’ pangenome by clustering the homologous gene sequences into gene families. PanTA^17^ is chosen as the method for pangenome construction for its speed and scalability among other pangenome inference methods such as Roary^14^ and Panaroo.^19^ PanTA can pre-build the pangenome from the training genomes, and during inference, can seamlessly integrate the test genomes into the existing pangenome with minimal additional computation with its progressive mode. This approach avoids the need to rebuild the entire pangenome from scratch at prediction time. The pangenome represents the entire set of genes within a species, and the presence of each gene in the genome of each isolate. A gene family is considered AMR gene if it contains at least one gene sequence that is annotated to be AMR gene. The core genes, that are defined to be those present in the majority of isolates, are extracted from the pangenome. The multiple alignments of all gene sequences in a gene family is also generated. This presentation enables PanKA to extract three sets of features that are relevant for AMR prediction: (1) the gene presence and absence matrix (namely PA matrix), (2) the protein k-mer profiles of AMR genes (AMR Kmer), and (3) the amino acid variants of core genes (PanCore). Next, to reduce training time and minimize the risk of overfitting, PanKA conducts feature selection to eliminate irrelevant and duplicate features in the amino acid variants, k-mer profiles, and presence and absence matrix. It filters out features with a low association with the target variable, as measured by Chi-squared statistics. The remaining features are then used to train a Light Gradient Boosting Machine (LightGBM) model.^23^ The main steps of the PanKA’s pipeline are given in Figure 1 and are described in detail in STAR Methods.

### Data and evaluation

We evaluated the performance of PanKA and compared with that of existing AMR prediction methods. Specifically, we compared the performance of PanKA to conventional k-mers based classifiers applied to whole DNA sequence (*KmerDNA*) and the protein sequence (*KmerProtein*), which are commonly used to predict AMR in the literature.^25^^,^^26^^,^^27^ Similar to PanKA, we applied the LightGBM model on the k-mer profiles of DNA and protein sequences. In addition, we compared PanKA to PanPred,^9^ the state-of-the-art classifier for AMR prediction using the pangenome. PanPred trained gradient boosted decision trees (GBDTs) and other machine learning algorithms on various types of predictors, including pangenome, population structure, isolation year, gene content, and polymorphism information. We found that the LightGBM classifier was more effective than all other machine learning algorithms used in PanPred for PanPred predictors. Thus for a fair comparison, we retrained the PanPred model using LightGBM (PanPred [LightGBM]). We also included the PanPred (default) as the original PanPred model trained with the GBDTs.

We evaluated PanKA and competing methods on two real datasets constructed from collections of genomes of two bacterial species, *E. coli* and *K. pneumoniae*. In particular, we examined 1653 strains from four extensive *E. coli* collections that had information on the drug susceptibility phenotypes, and whole genome sequences.^28^^,^^29^ The antibiotics include penicillin: ampicillin (AMP); cephalosporins: cefuroxime (CXM), cefotaxime (CTX), cephalothin (CET) and ceftazidime (CAZ); aminoglycosides: gentamicin (GEN), tobramycin (TBM); and fluoroquinolones: ciprofloxacin (CIP); and amoxicillin-clavulanate (AMC), amoxicillin (AMX) and trimethoprim (TMP). AMR phenotypes (susceptible, intermediate, and resistant) were obtained from the previous study.^9^

The *K. pneumoniae* datasets were downloaded from the Pathosystems Resource Integration Center (PATRIC) database, one of the most comprehensive public databases of bacterial genomes and AMR metadata. The antibiotics for *K. pneumoniae* dataset include ampicillin-sulbactam (AMS), aztreonam (AZT), cefazolin (CZL), cefepime (FEP), cefoxitin (FOX), ceftazidime (CAZ), ceftriaxone (CTR), cefuroxime sodium (CXM), CIP, gentamicin (GEN), imipenem (IPM), levofloxacin (LVX), nitrofurantoin (NIT), piperacillin-tazobactam (TZP), tetracycline (TCY), tobramycin (TOB), TMP/sulfamethoxazole (SXT). We collected all strains on PATRIC that have the whole genomes and the corresponding phenotypic information (susceptible, intermediate, and resistant) available in the database. Consistent with PanPred, isolates associated with the intermediate phenotype were considered as resistant, creating two groups of phenotypically distinct isolates, i.e., susceptible and non-susceptible. The number of resistant and susceptible isolates for the two datasets is presented in Tables 1 and 2.

**Table 1:** Phenotype information of the *E. coli* dataset

Drugs	AMC	AMP	AMX	CET	CIP	CTX	CAZ	CXM	GEN	TBM	TMP
Resistant	501	395	659	205	358	264	143	388	214	144	245
Susceptible	1151	167	431	357	1294	1312	1509	1264	1438	418	317
Total	1652	562	1090	562	1652	1576	1652	1652	1652	562	562

The number of resistant and susceptible samples on the *E. coli* dataset.

**Table 2:** Phenotype information of the *K. pneumoniae* dataset

Drugs	AMS	AZT	CZL	FEP	FOX	CAZ	CTR	CXM	CIP	GEN	IPM
Resistant	1620	1638	1772	1136	998	1902	1741	1533	1885	952	676
Susceptible	91	229	197	575	856	149	178	93	354	1244	1283
Total	1711	1867	1969	1711	1854	2051	1919	1626	2239	2196	1959

Drugs	LVX	NIT	TZP	TCY	TOB	SXT
Resistant	1509	820	1222	841	994	1651
Susceptible	377	93	575	771	758	567
Total	1886	913	1797	1612	1752	2218

The number of resistant and susceptible samples on the *K. pneumoniae* dataset.

All classifiers were built to predict two distinct AMR phenotypes: “resistant” vs. “susceptible”. Consistent with the benchmark in PanPred,^9^ the dataset was randomly split, with 80% designated for training and model optimization purposes. We performed hyperparameter tuning using the mean F1 score across 5-fold cross-validation as the objective function. For a fair comparison, we applied GridSearchCV in scikit-learn library to fine-tune the same set of parameters for all methods. Specifically these parameters include the maximum number of bins used during the feature value discretization process (max_bin) with values of 2, 3, 4, and 8; the maximum depth of the tree (max_depth) with values of 5, 7, 9, and 11; the minimum number of samples in a leaf (min_child_samples) with values of 5 and 20; and the maximum number of leaf nodes in a tree (num_leaves) with values of 31 and 63. Subsequently, we evaluated the performance of the trained model on the remaining 20% of the dataset, which served as the held-out test data. The accuracy of the predictions was measured using the F1-score, which combines precision and recall into a single score that balances both metrics. To confirm statistical significance, we performed the Wilcoxon signed-rank test to compare the differences between methods, using a significance level of 0.05.

### Predicting AMR phenotypes using PanKA

**Figure 2:**
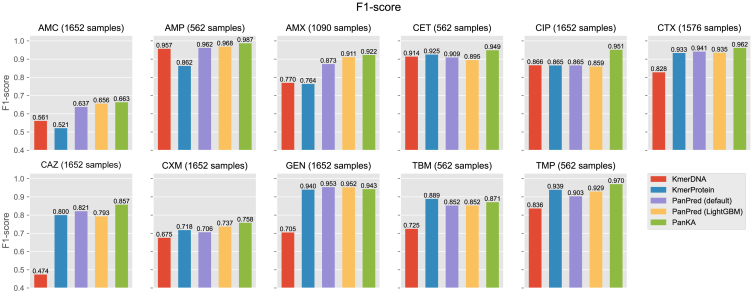
Prediction performance on the *E. coli* dataset The classification performance of resistance prediction on the *E. coli* datasets is illustrated in a bar plot, which displays the F1-score for each method on a test set consisting of 20% of the samples. PanKA is a combination of 3 features: PanCore, AMR Kmer and PA matrix. KmerDNA refers to applying LightGBM to k-mer features extracted from the whole DNA sequence, while KmerProtein refers to applying LightGBM to k-mer features of protein-coding gene sequences. PanPred (default) trained gradient boosted decision trees (GBDT), and PanPred (LightGBM) retrained the PanPred model using LightGBM.

We initially compared all methods using the *E. coli* datasets. As shown in Figure 2, PanKA demonstrates superior performance overall, especially for the AMC, CET, CIP, CAZ, CMX, and TMP phenotypes. In contrast, the prediction models trained on the k-mer profiles of DNA (KmerDNA) and protein (KmerProtein) exhibited the lowest performance. Meanwhile, PanKA achieved more accurate phenotyping than the competing methods in almost all phenotypes. On 9 out of 11 types of antibiotics, PanKA achieved higher F1 scores than the best competing method, PanPred, except for the CAZ and GEN phenotypes where PanPred performed marginally better than PanKA. Specifically, the average F1 score of PanKA across all phenotypes is 0.893, significantly higher than that of PanPred (default) at 0.856 (*p* value < 0.003). The precision and recall metrics further underscored PanKA’s efficacy, aligning closely with the F1 scores across most phenotypes (Figure S1). The average recall of PanKA is 0.872 while that of PanPred is 0.826 (*p* value < 0.006). However, notable discrepancies were observed, particularly concerning the AMC and CAZ phenotypes. In the case of the AMC phenotype, competing methods displayed marginally higher precision than PanKA, albeit at the expense of lower recall. Furthermore, while KmerDNA achieved perfect precision for the CAZ phenotype, its recall was considerably lower. Of particular interest is the observation that PanPred, when trained with the LightGBM model yielded an average F1 score of 0.862, generally performed better than its default version with an average F1 score of 0.856. While LightGBM provides only a marginal improvement over PanPred’s default model using GBDT, it offers significantly faster computational speed compared to GBDT.

We proceeded to dissect the distinct contributions of three PanKA modalities in PanKA’s performance. Specifically, we ran LightGBM on the AMR Kmer features extracted by PanKA, denoted as PanKA (*AMR Kmer*). Upon analysis, the performance of PanKA marginally surpassed that of PanKA (*AMR Kmer*), with the exception of the GEN, and TBM phenotypes (Figure S2). Notably, for the CIP and TMP antibiotics, PanKA demonstrated significantly higher metrics than PanKA (AMR Kmer), indicating the importance of incorporating PanCore and PA matrix features in these phenotypes.

Additionally, we explored an alternative method for feature extraction within the AMR genes. Instead of using k-mer profiles, we considered using amino acid variants of the AMR genes (referred to as *AMR AVs*), similar to how amino acid variants of core genes (PanCore) are used. As shown in Figure S2, employing AMR Kmer profiles (PanKA [AMR Kmer]) generally produced better results for predicting resistance compared to using amino acid variants in AMR genes (PanKA [AMR AVs]). Even when combining amino acid variants of the AMR genes (AMR AVs) with other features (referred to as PanKA [AMR AVs + PA matrix + PanCore]), the performance remained lower than that of PanKA (Figure S2). PanKA performs better than PanKA (AMR AVs + PA matrix + PanCore) across all three metrics. Specifically, PanKA achieves a higher F1 score than PanKA (AMR AVs + PA matrix + PanCore) (0.893 vs. 0.877), indicating better overall performance. PanKA also demonstrates higher precision (0.923 vs. 0.918) and recall (0.872 vs. 0.846) than PanKA (AMR AVs + PA matrix + PanCore). In addition, PanKA demonstrates statistically significant improvement over PanKA (AMR AVs + PA matrix + PanCore), with a *p* value < 0.05 by the Wilcoxon signed-rank test. This result supports the conclusion that PanKA’s enhancements lead to a measurable and meaningful increase in predictive accuracy compared to its version with AMR AVs.

Next, we investigated the effect of varying k-mer sizes in AMR Kmer on the performance of PanKA (Figure S3). Specifically, we evaluated PanKA using k-mer sizes of k=5,8,10,12,15, and 20. The results indicated that, with the exception of k=5 which exhibited slightly lower performance compared to the other k-mer sizes, PanKA maintained robust performance across the different k-mer values. The small standard deviations of performance metrics (F1, precision, recall) across all k-mer sizes (std < 0.02) underscored the stability and robustness of PanKA with respect to varying k-mer sizes.

**Figure 3:**
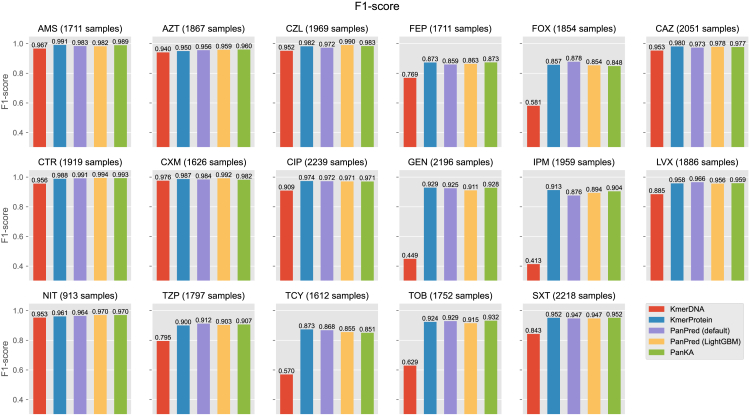
Prediction performance on the *K. pneumoniae* dataset The classification performance of resistant prediction on the *K. pneumoniae* datasets is depicted in a barplot, similar to the *E. coli* datasets. KmerDNA refers to applying LightGBM to k-mer features extracted from the whole DNA sequence, while KmerProtein refers to applying LightGBM to k-mer features of protein-coding gene sequences. PanPred (default) trained gradient boosted decision trees (GBDT), and PanPred (LightGBM) retrained the PanPred model using LightGBM.

For the *K. pneumoniae* datasets, it can be seen in Figures 3 and S4 that most methods achieved scores close to 1. Specifically, the F1 scores of PanKA were slightly better than those of PanPred and *KmerProtein*. Consistent with the *E. coli* datasets, *KmerDNA* recorded the lowest F1 score. This pattern is consistently reflected in the precision and recall metrics (Figure S4). The results suggest that PanKA is a promising method for predicting AMR in *K. pneumoniae* datasets. However, it is important to note that the performance of PanKA and other methods may vary depending on the dataset and the specific AMR mechanism being studied.

### Evaluation of the models based on sequence type stratification

We investigated the population structure of pathogens and evaluated the model’s performance stratified by sequence types (ST) using the *E. coli* datasets. Population structure could potentially impact the accuracy of our models. To obtain ST, we employed multi-locus sequence typing using the tools available at https://github.com/tseemann/mlst. For each antibiotic, we calculated the ratio of resistant ST isolates to the total number of ST isolates. As shown in Figure S5, our analysis revealed that no resistant phenotype was exclusively associated with a single ST. It can be seen that each resistant phenotype comprises multiple subtypes, with certain subtypes appearing consistently across the majority of phenotypes.

Following our initial analysis, we proceeded to evaluate the model’s performance based on ST. To achieve this, we stratified the train and test sets by ST, ensuring that all isolates of a given ST were exclusively assigned to either the training or test sets. We varied the ST and the number of types to generate five distinct test sets: test set 1 (ST131 and ST95), test set 2 (ST10, ST127, ST405, ST38, ST393, ST14, ST80, ST62, ST59, ST357, ST141, ST648, and ST58), test set 3 (ST131, ST12, ST10, and ST127), test set 4 (ST95, ST10, ST405, ST38, ST393, ST88, ST144, ST14, and ST80), and test set 5 (ST131 and ST69). Additionally, the test sets were designed to maintain a test size of approximately 20% of the samples. The results of our analysis, as presented in Figure S6, indicate no significant decline in the precision, recall, and F1 score of PanKA when compared to the non-stratified settings. This is evident as most of PanKA’s scores in the non-stratified settings (PanKA with random split, shown by the dashed line) fall below the median of the boxplot (PanKA with sequence type stratification, with the median represented by the mid-line of the boxplot). However, there is a notable exception for the CAZ and TMP phenotypes, where PanKA’s performance was lower than in previous experiments. These findings suggest that the various subgroups of *E. coli* possess similar resistant mechanisms, and our markers (features) are predominantly determined by resistance/susceptibility rather than ST. Notably, this conclusion aligns with previous research findings.^9^^,^^30^

### Marker analysis

**Figure 4:**
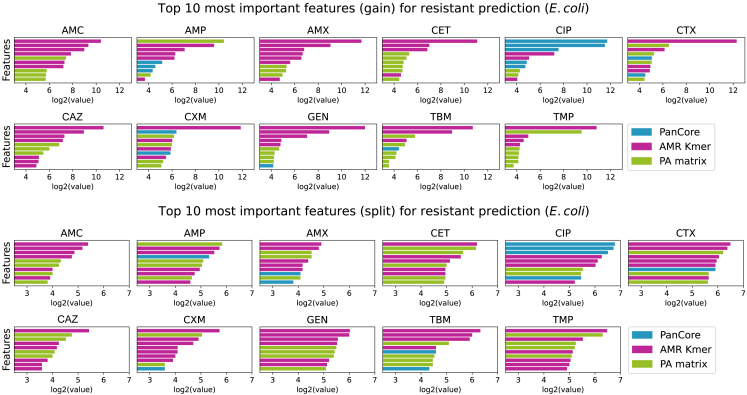
Feature ranking on the *E. coli* dataset Feature ranking (top 10) by “gain” score (top) and “split” score (bottom) for predicting antibiotics on the *E. coli* dataset. The “gain” score represents the improvement in the objective function resulting from adding a split point to a tree node. Higher gain indicates that the feature provides more significant information for making predictions. The “split” score is defined as the number of times a feature is used to split the tree. A higher split score indicates that the feature is frequently used to make decisions in the model.

**Figure 5:**
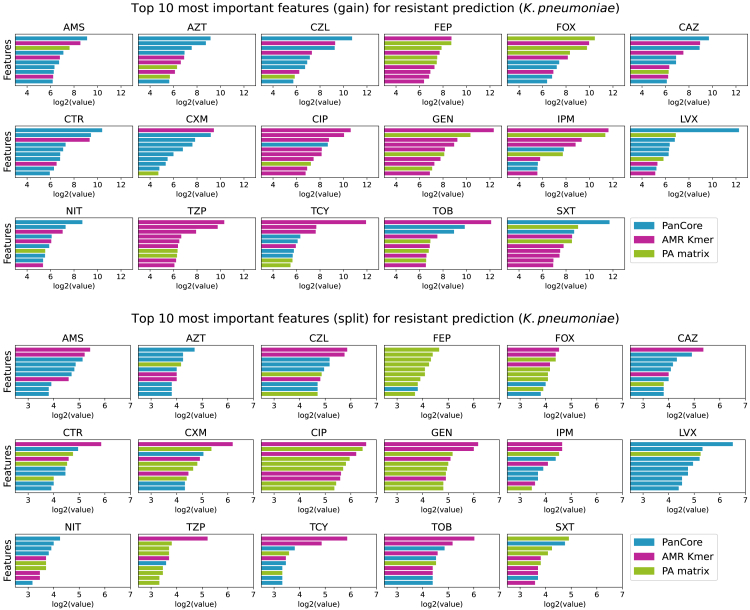
Feature ranking on the *K. pneumoniae* dataset Feature ranking (top 10) by “gain” score (top) and “split” score (bottom) for predicting antibiotics on the *K. pneumoniae* dataset. The “gain” score represents the improvement in the objective function resulting from adding a split point to a tree node. Higher gain indicates that the feature provides more significant information for making predictions. The “split” score is defined as the number of times a feature is used to split the tree. A higher split score indicates that the feature is frequently used to make decisions in the model.

Finally we dissect the importance of predictors in the machine learning model. In particular, we investigate the “gain” and “split” scores which are two main measures of feature importance in LightGBM. The split score measures the number of times a certain feature is used for splitting the data across all trees in the model, whereas the gain score qualifies the improvement of model accuracy achieved by using the particular feature for splitting. The top 10 gain and split scored features for *E. coli* and *K. pneumoniae* are shown in Figures 4 and 5, respectively. Detailed annotations of these markers, including Prokka gene ID, protein function, and position within the gene cluster, are available in Tables S1, S2, S3, and S4. Specifically, Tables S1 and S2 provide the annotation of the top 10 features for *E. coli* based on “gain” and “split” scores, respectively, while Tables S3 and S4 offer the annotation of the top 10 features for *K. pneumoniae* based on “gain” and “split” scores, respectively. It can be seen that all three types of predictors (the AMR Kmer, PA matrix, and PanCore) used in PanKA are frequently appeared in the top features, highlighting the importance of using them in the model. The contribution of each feature type is, however, different between two datasets. In the *E. coli* dataset, the AMR Kmer and the presence and absence of genes are primarily important features while the PanCore (amino acid variants in the pan-genes) features are the most frequently used. This also explains why the performance of PanKA is similar to PanPred for the *K. pneumoniae* dataset as they both employ the PA matrix and PanCore.

We found that there is little overlap between the top features reported by gain and by split scores, possibly due to the difference in how the features are ranked. In examining the most AMR predictive features identified by both scores (Tables S1, S2, S3, and S4), we found about over half of the features were in annotated genes. Strikingly, most of these annotated genes are known to be associated with antibiotic resistance. For example, in the top 10 gain-based and 10 split-based features for AMC resistance in *E. coli*, the annotated features are in genes *oleI*, *emrB*, *blaTEM*, *aadA5*, *ileS*, *ugpB*, *atoC*, *ddpF*, *lsoA*, *fdtC*, *lsoA*, *hlyB*, *FosA*, and *tolA*, all of them were known to be resistant genes. It would be interesting to study the unannotated features to gain insights into their association with AMR as identified by PanKA. This also demonstrates the interpretability of PanKA.

### Time and memory usage

We demonstrate the scalability of PanKA to large datasets. Table 3 presents the average CPU times (in minutes) and peak memory usage (in GB) for training and testing the methods on the real datasets from the two bacterial species used in this study. Since all methods but *KmerDNA* required the annotation of genomes, we excluded the running times of the genome annotation using Prokka. It can be shown that the runtime of PanKA (training + testing) was around 20 min, which was about 8 times faster than the second fastest method, PanPred (LightGBM). *KmerDNA* was the slowest, taking approximately four days to complete. Table 3 (right) shows the peak memory usage for the same simulated datasets. PanKA required the least amount of memory (<4 GB), followed by PanPred (>18 GB). *KmerDNA* and *KmerProtein* used significantly more memory (>150 GB) than the other methods.

**Table 3:** Running times analysis

	Runtime (minutes)		Memory (Gb)	
	*E. coli*	*K. pneumoniae*	*E. coli*	*K. pneumoniae*
PanKA	20.1	24.9	3.3	3.6
PanPred	170.8	223.1	18.4	20.3
KmerDNA	5567.1	5976.6	217.1	233.4
KmerProtein	3400.2	4536.1	150.3	160.3

Comparison of running times and peak memory usage on real datasets. Average running times (in minutes) and memory usage (in GB) of PanKA, PanPred, KmerDNA, and KmerProtein are reported on the real datasets used in this study. PanKA: Our proposed method, combining PanCore, AMR Kmer, and PA matrix features. PanPred: A state-of-the-art method for predicting antibiotic resistance. KmerDNA: A method that applies LightGBM on k-mer features extracted from the whole DNA sequence, encompassing coding and non-coding regions. KmerProtein: A method that uses LightGBM on k-mer features derived from protein-coding sequences only.

## Discussion

The use of machine learning-based methods for predicting AMR has gained popularity recently, as they have achieved promising results without requiring any prior knowledge of AMR mechanisms. We have introduced PanKA, a method that predicts AMR phenotypes by performing the state-of-the-art classifier LightGBM on the AMR-relevant features extracted from the pangenome. We have demonstrated that PanKA combines multiple features from the data and exploits relationships among them to better predict AMR phenotypes. We showed that our approach produces more accurate and robust predictions of AMR compared to conventional methods of counting k-mers and the state-of-the-art classifier for the AMR predictions.

AMR in bacteria can arise through two important types of genetic mechanisms: mutation and acquisition of new genetic materials. In the case of mutation, the rate at which resistance develops can be attributed to the rate at which bacteria mutate. Acquisition of new genetic materials can occur through horizontal gene transfer, which is the transferring of genetic materials between different organisms that are not parent and offspring. The features constructed in PanKA, including the presence and absence of genes, amino acid variants, and k-mers, aim to capture the two genetic mechanisms of antibiotic resistance. The PA matrix captures the presence and absence of AMR genes, while the amino acid variants and k-mer counts account for bacterial mutations.

The concept of using a population genome graph as a comprehensive and compact reference model has been proposed in various applications of human and microbial genomics.^31^^,^^32^^,^^33^^,^^34^^,^^35^ This technique has been shown to have advantages over the traditional linear reference in alignment, variant calling, and sequence typing analyses. A pangenome can be constructed from a set of bacterial genome assemblies using tools such as Roary,^14^ Panaroo,^19^ or PanTA.^17^ PanKA uses the pangenome to represent population of bacterial genomes, which reduces bias toward a single reference genome. Our method extracts only a limited number of features that are pertinent to AMR, resulting in faster training times and cost savings. Given the rapid evolution of bacterial genomes, it may be necessary to retrain the machine learning model regularly.

### Limitations of the study

The phenotype data in our benchmarks is provided as a binary label, categorizing each isolate as either resistant or susceptible to an antibiotic. To predict multi-drug resistant (MDR) pathogens, a strategy involving the combination of all binary classifiers could be employed. However, it is plausible that consolidating antibiotics into a single model may enhance accuracy by leveraging support from related antibiotics. It is worth noting that one could use multi-label classification techniques to predict MDR pathogens directly. Validating the performance of such multi-label classification models on real MDR pathogens would be a valuable next step. However, it should be acknowledged that the combination of models may not significantly impact the overall performance of the model.^30^

In addition to simply categorizing strains as resistant or susceptible, there is potential to predict the minimum inhibitory concentration (MIC) range of strains using available whole-genome sequences. MIC prediction involves quantitatively estimating the susceptibility of bacterial strains to specific antibiotics. By harnessing advanced computational methods and comprehensive genomic data, models capable of predicting MIC ranges based on genomic features using machine learning, such as regression models, could be developed. Exploring this path of incorporating MIC levels would be a crucial area for future investigation.

In addition, we plan to validate our method on larger datasets and investigate the top AMR-associated features from the machine learning model. This will help us to further improve the accuracy of our predictions and identify the most important features that contribute to AMR. We believe that PanKA will provide an accurate method to predict AMR, identify antimicrobial mechanisms, and combat the spread of AMR.

## STAR★Methods

### Key resources table

**Table:** 

REAGENT or RESOURCE	SOURCE	IDENTIFIER
**Software and algorithms**		

PanPred: Prediction of antibiotic resistance in Escherichia coli from large-scale pan-genome data	PanPred	https://doi.org/10.1371/journal.pcbi.1006258
PanKA	This paper	https://zenodo.org/doi/10.5281/zenodo.10966778

### Resource availability

#### Lead contact

Further information and requests for resources should be directed to and will be fulfilled by the lead contact, Dr. Minh Duc Cao (minhduc.cao@gmail.com).

#### Materials availability

This study did not generate new biological data.

#### Data and code availability

• We have compiled the metadata for the *E. coli* and *K. pneumoniae* datasets, which include the European Nucleotide Archive (ENA) accession numbers and Patric ID, along with their respective resistance profiles. This comprehensive information is provided in Tables S5 and S6.
• All original code has been deposited at Github (https://github.com/amromics/panka) and is publicly available as of the date of publication. DOI is listed in the key resources table.
• Any additional information required to reanalyze the data reported in this paper is available at Github (https://github.com/amromics/panka).

### Experimental model and study participant details

This paper analyzes existing, publicly available data. The study does not use experimental models typical in life sciences.

### Method details

#### Construction of the AMR pangenome

The genome assemblies are first annotated with Prokka.^24^ Prokka aims to identify protein coding regions in the genome using Prodigal^36^ and then predicts the function of the encoded protein by comparing it with proteins in various protein databases. In addition, Prokka identifies AMR genes using the NCBI Bacterial Antimicrobial Resistance Reference Gene Database. The annotated assemblies are then used as input for PanTA^17^ to build a pangenome.

PanTA outputs homologous gene groups and a matrix indicating the presence and absence of gene clusters. Gene clusters that contain genes from at least 65% of the strains are referred to as *pan-genes*. PanTA then produces a multiple protein alignment of amino acid sequences for each cluster in the pan-genes using mafft.^37^ In addition, a gene cluster with at least one AMR gene is referred to as *AMR gene cluster*. The pan-genes, AMR gene clusters, and the presence and absence of genes matrix are used as features to build the LightGBM model.

#### Feature engineering from the pangenome

First, a matrix of amino acid variants is extracted from the multiple protein alignment of amino acid sequences in pan-genes (*PanCore*). PanKA employs a label encoder to encode the amino acids in the alignment. Each amino acid is assigned a unique integer from 1 to 20, representing the 20 types of amino acids, and 0 represents gaps.

Next, PanKA uses k-mer profiles (presence and absence of k-mers) (k = 10) for each protein sequence within an AMR gene cluster (termed AMR Kmer). For DNA analysis, the choice of k = 31 has been optimal for various k-mers based antimicrobial resistance (AMR) prediction methods,^25^^,^^26^^,^^38^ benchmarks,^39^^,^^40^ and is also the maximal value supported by certain k-mer count tools.^41^ Therefore, for protein sequences, we opted for k = 10. This decision was influenced by the fact that three DNA nucleotides encode one amino acid in proteins. Additionally, selecting k = 10 strikes a balance between sequence representation and computational efficiency. While a larger k value could potentially capture more intricate sequence patterns, it would also result in a significantly larger number of features, necessitating substantial computational resources. In addition, our experiments showed that the PanKA’s performance is robust to the choice of *k* (Figure S3). Based on our experiments, utilizing AMR Kmer profiles yields better results for predicting resistance compared to encoding amino acid variants present in AMR genes. The k-mer profiles can capture genetic variants by identifying the frequency of k-mers.^42^ These variants can be single nucleotide polymorphisms (SNPs), insertions, deletions, or structural variations. Subsequently, the PanCore, AMR Kmer, and the presence and absence of genes matrix (termed *PA matrix* hereafter) are combined as the input for the machine learning model.

#### Feature selection and machine learning models

Due to the large number of features in the data, PanKA performs feature selection and filtering to reduce the number of spurious features. Specifically, PanKA employs the Chi-squared test for feature selection, aimed at eliminating irrelevant features within PanCore, AMR Kmer, and PA matrix. The Chi-squared test measures the association between categorical features (predictors) and the target variable. It involves calculating the Chi-squared statistic and p-value, and selecting features with high Chi-squared statistics, which indicates a strong association with the target variable. Specifically, PanKA computes the Chi-squared statistics for all features for each antibiotic. Subsequently, it uniformly selects the top 2,000 features (with the largest Chi-squared statistics) for each feature type (PanCore, AMR Kmer, and PA matrix). This selection criterion is chosen to strike a balance between computational complexity and efficiency.

PanKA employs LightGBM^23^ (Light Gradient Boosting Machine), a distributed gradient-boosting framework for machine learning. It is based on decision tree algorithms and used for ranking, classification, and other machine learning tasks. Compared to other popular decision tree algorithms such as Gradient Boosting Decision Tree and XGBoost, LightGBM employs a leaf-wise tree growth strategy, which provides several advantages. These include faster training speed with lower memory usage, improved accuracy and capable of handling large-scale data. LightGBM has gained tremendous popularity among machine learning practitioners due to its speed and accuracy.^43^^,^^44^^,^^45^ In addition, LightGBM reports the feature importance in terms of “gain” and “split” score (see LightGBM documentation^23^^,^^46^). The gain and split scores are two important metrics used in the LightGBM algorithm. The gain is the improvement in the objective function that results from adding a split point to a tree node. The split score is defined as the number of times that a feature is used to split the tree.

### Quantification and statistical analysis

#### Evaluation metrics

The accuracy of the predictions was measured using the F1-score, which combines precision and recall into a single score that balances both metrics. We also calculated precision and recall for all models. Generally, a higher score signifies better performance.

#### Statistical analysis

We conducted a Wilcoxon signed-rank test to compare the differences between methods, using a significance level of 0.05.

## Supplemental information

Document S1. Figures S1‒S6

Table S1. *E. coli* gain feature annotation

Table S2. *E. coli* split feature annotation

Table S3. *K. pneumoniae* gain feature annotation

Table S4. *K. pneumoniae* split feature annotation

Table S5. List of isolates of *E. coli* dataset

Table S6. List of isolates of *K. pneumoniae* dataset
