# Supplementary material for: PanKA: Leveraging population pangenome to predict antibiotic resistance
Source: iScience. 2024 Aug 2;27(9):110623. doi: 10.1016/j.isci.2024.110623 (PMC11369404; doi:10.1016/j.isci.2024.110623)
Supplement: Document S1. Figures S1‒S6 [file mmc1.pdf]

iScience, Volume *27*

Supplemental information

PanKA: Leveraging population pangenome to predict antibiotic resistance

Van Hoan Do, Van Sang Nguyen, Son Hoang Nguyen, Duc Quang Le, Tam Thi Nguyen, Canh Hao Nguyen, Tho Huu Ho, Nam S. Vo, Trang Nguyen, Hoang Anh Nguyen, and Minh Duc Cao

Supplemental Information: PanKA: Leveraging population pangenome to predict antibiotic resistance.

1. Supplemental Figures

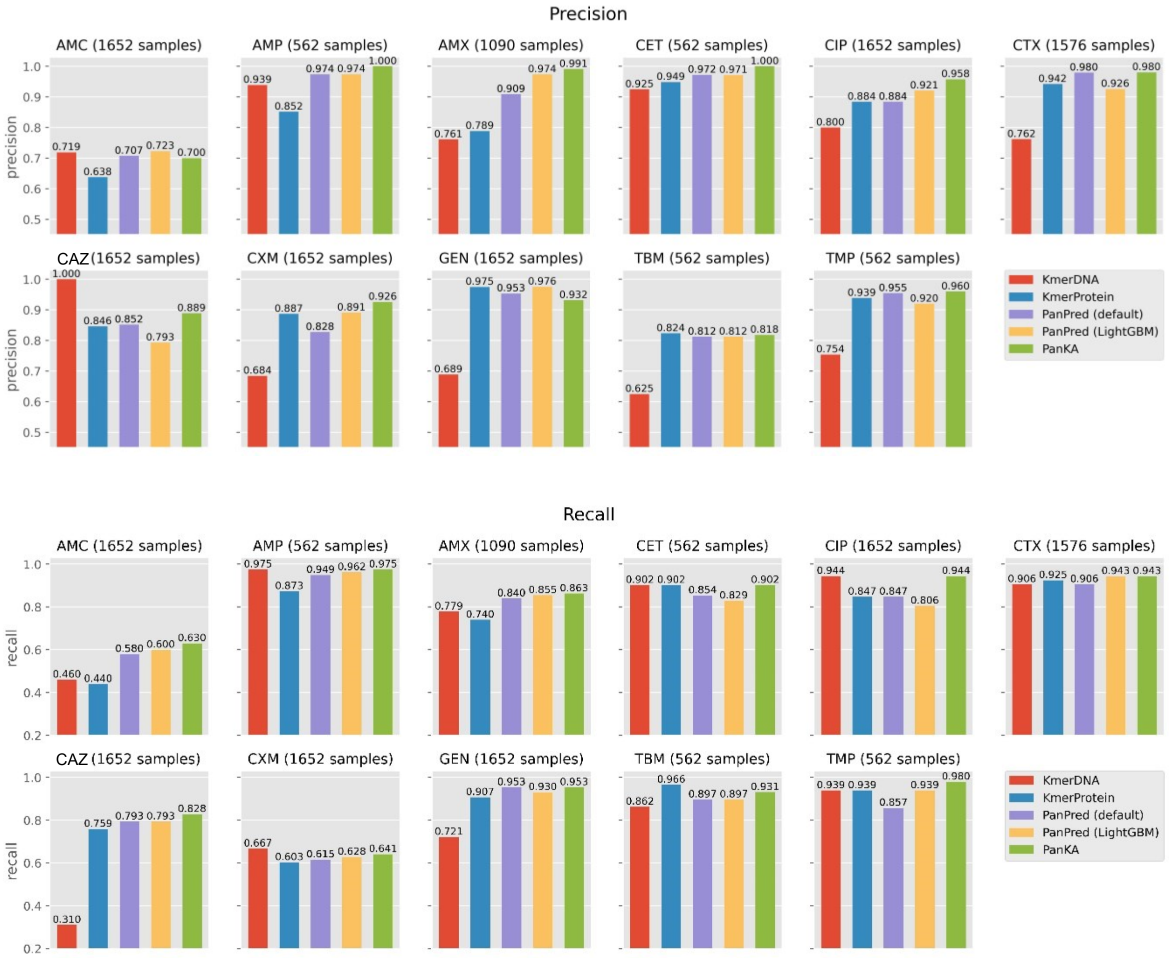

Figure S1. Prediction performance in the *E. coli* dataset
The precision (*top*) and recall (*bottom*) of PanKA and competting methods on the *E. coli* datasets. PanKA integrates PanCore, AMR Kmer, and PA matrix features to optimize predictive accuracy. Competing methods include PanPred, which employs gradient boosted decision trees (GBDT) in default and LightGBM-retrained configurations, as well as KmerDNA and KmerProtein focusing on k-mer features extracted from whole DNA and protein-coding sequences, respectively.

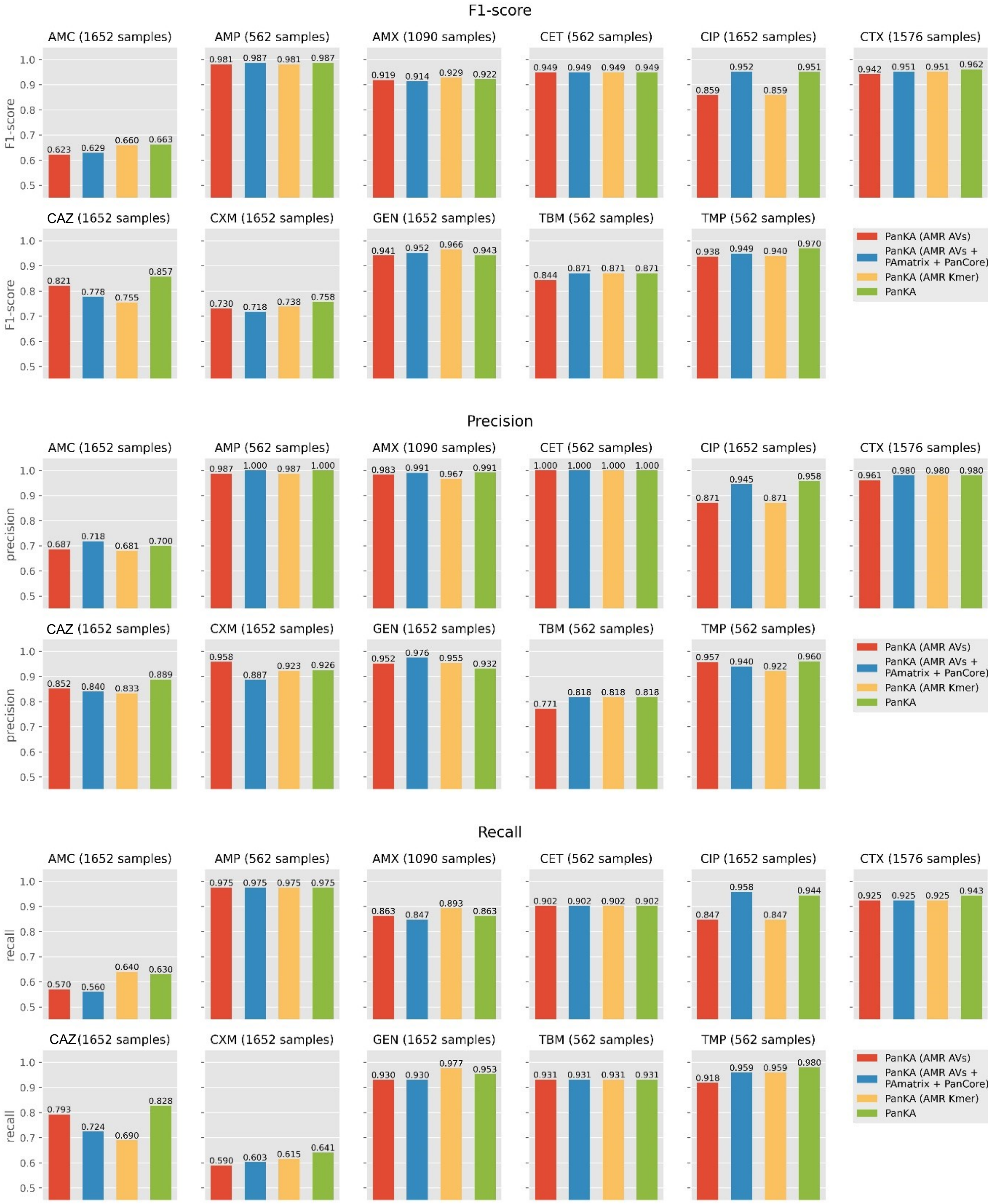

Figure S2. Prediction performance of PanKA and its variation

The F1 score (*top*), precision (*middle*) and recall (*bottom*) of PanKA and its variation on the *E. coli* datasets. The features used for training the model are in the parentheses. AMR AVs: amino acid variants extracted in AMR gene clusters, PA matrix: the presence and absence of genes matrix, AMR Kmer: k-mers extracted from AMR genes. PanCore: amino acid variants extracted in core genes.

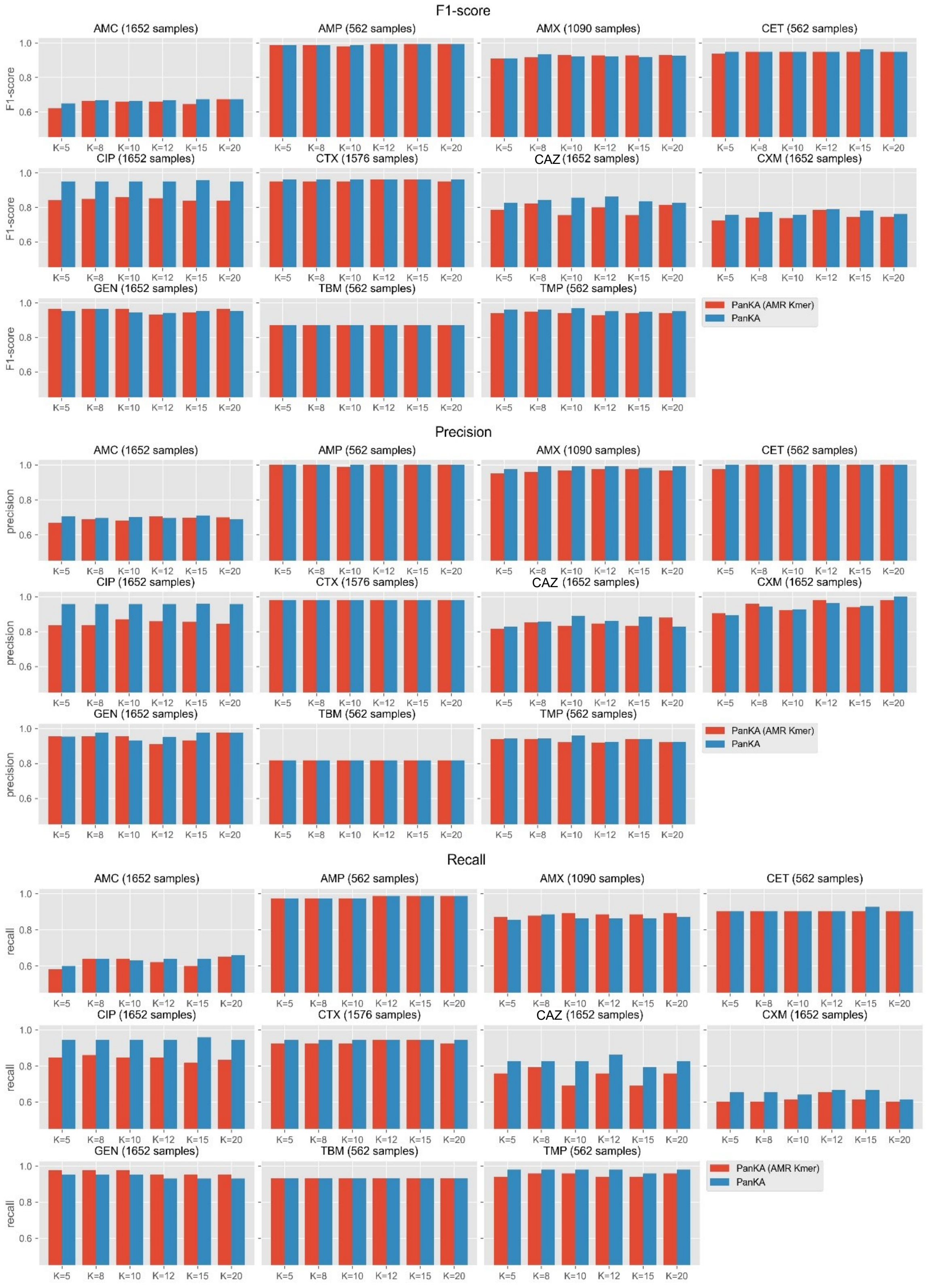

Figure S3. The performance of PanKA across various k-mer sizes. This shows PanKA's robustness and sensitivity to parameter variations. The plot depicts metrics such as F1 score, precision, and recall for different k values, including 5, 8, 10, 12, 15, and 20.

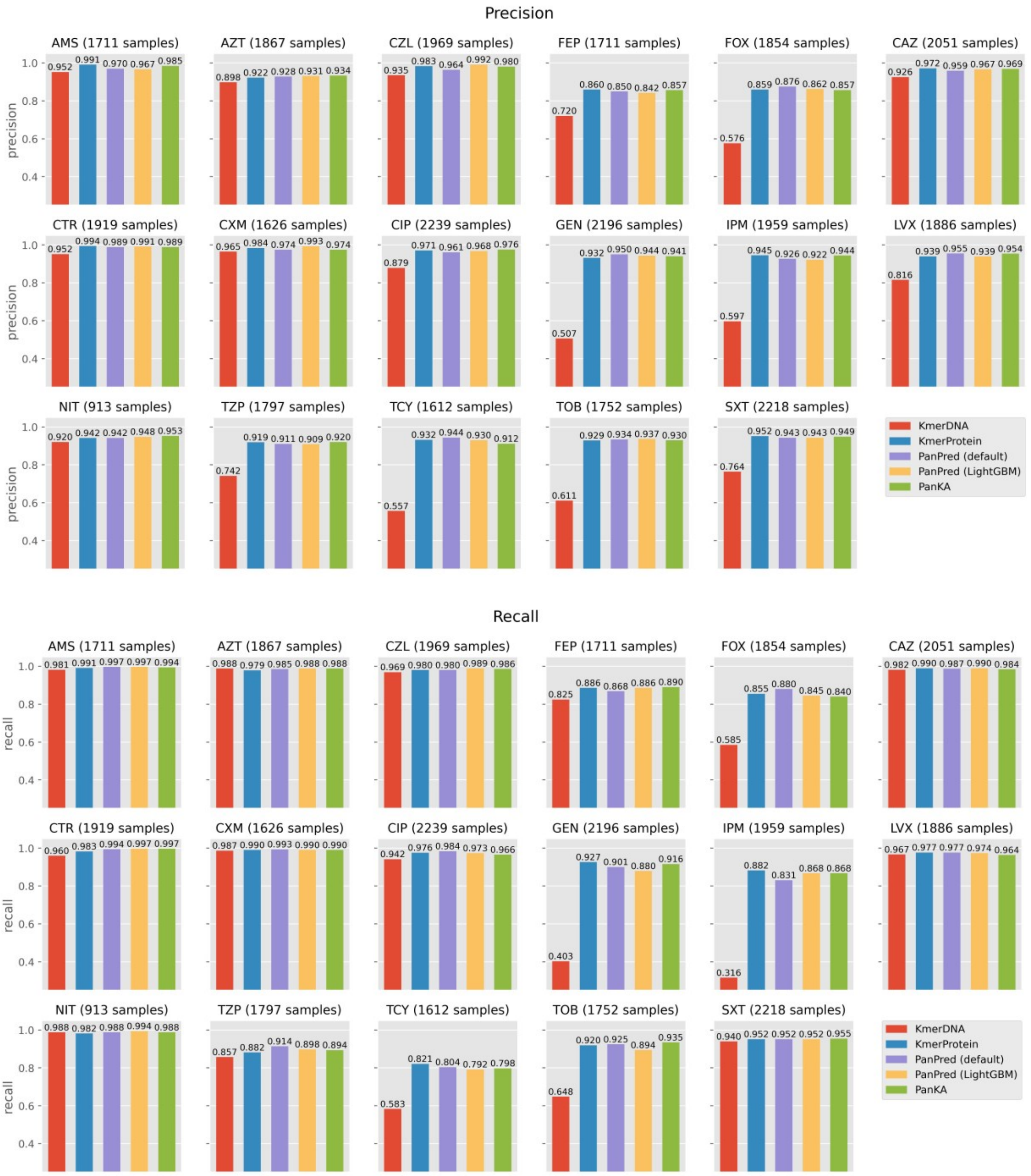

Figure S4. Prediction performance on the *K. pneumoniae* dataset

The precision (*top*) and recall (*bottom*) of PanKA and competting methods on the *K. pneumoniae* datasets. PanKA: Our proposed method, combining PanCore, AMR k-mer, and PA matrix features. PanPred: A state-of-the-art method for predicting antibiotic resistance. KmerDNA: A method that applies LightGBM on k-mer features extracted from the whole DNA sequence, encompassing coding and non-coding regions. KmerProtein: A method that uses LightGBM on k-mer features derived from protein-coding sequences only.

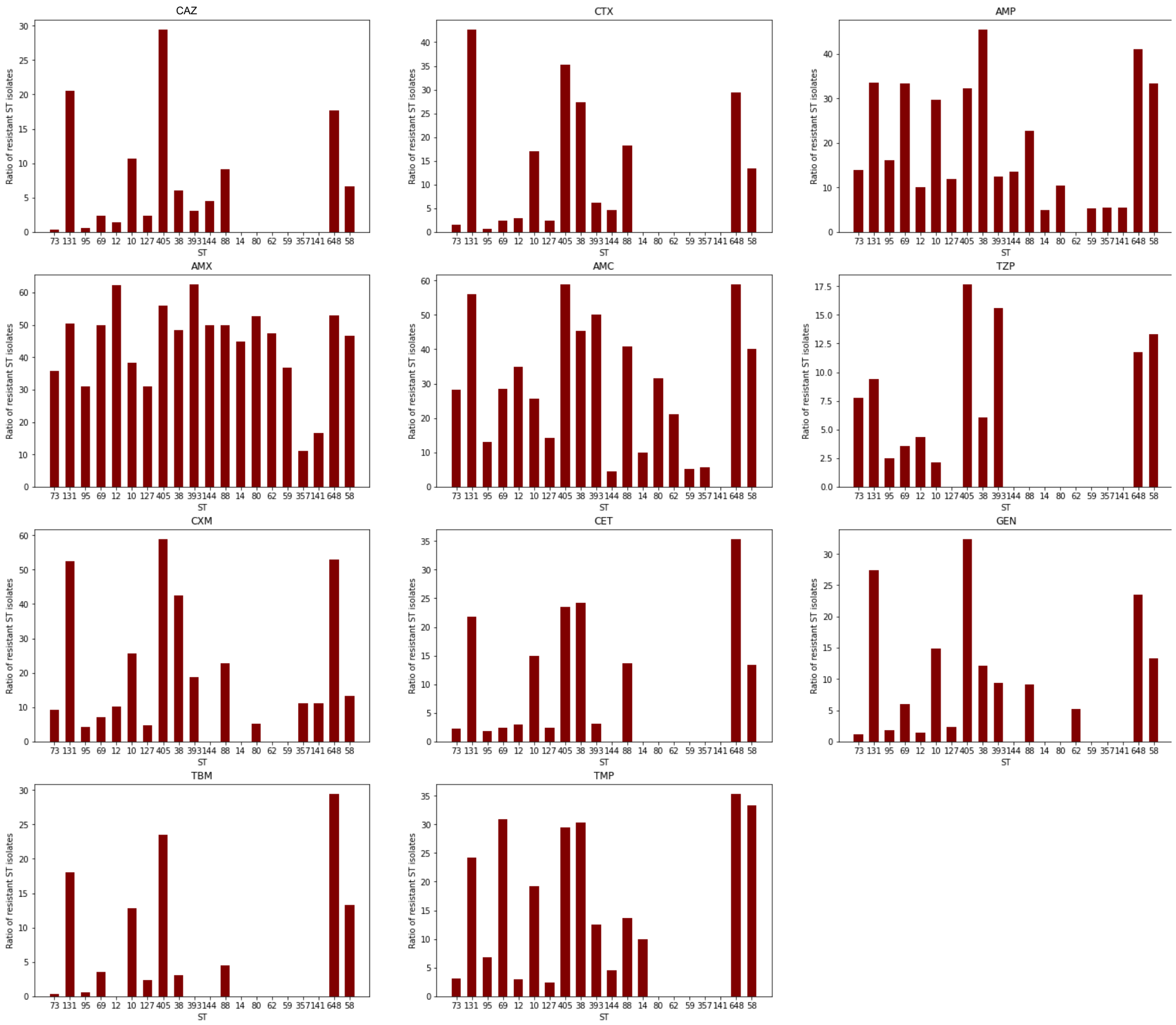

Figure S5. Distribution of sequence types for each antibiotic

The bar plot illustrates the ratio of resistant ST (sequence type) isolates to the total number of ST isolates. Each bar on the x-axis corresponds to a sequence type, showing how different antibiotics encompass various sequence types. This visualization emphasizes whether certain sequence types are predominantly associated with a specific phenotype within each antibiotic category.

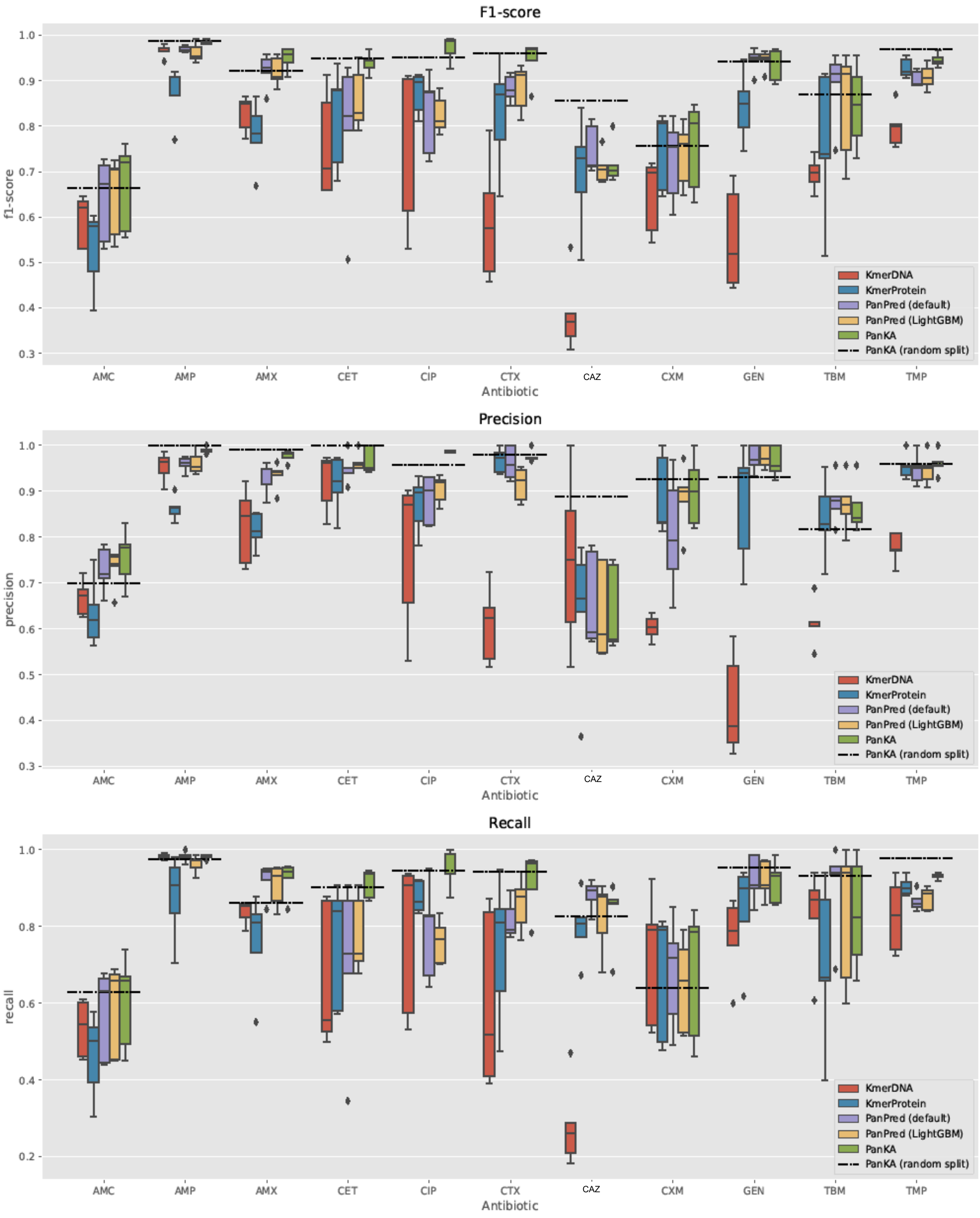

Figure S6. Prediction performance based on sequence type stratification
The boxplot of F1 score (*top*), precision (*middle*) and recall (*bottom*) of PanKA and competting methods on the *E. coli* datasets based on sequence type stratification. The horizontal dashed lines indicate the scores of PanKA in non-stratified settings (random split, see Figures 2 and S1). PanKA: Our proposed method. KmerDNA: A method that applies LightGBM on k-mer features extracted from the whole DNA sequence, encompassing coding and non-coding regions. KmerProtein: A method that uses LightGBM on k-mer features derived from protein-coding sequences only.
